# Optimization of an Extraction Process to Obtain a Food-Grade Sulforaphane-Rich Extract from Broccoli (*Brassica oleracea* var. *italica*)

**DOI:** 10.3390/molecules26134042

**Published:** 2021-07-01

**Authors:** Francis González, Julián Quintero, Rodrigo Del Río, Andrea Mahn

**Affiliations:** 1 Departamento de Ingeniería Química, Facultad de Ingeniería, Universidad de Santiago de Chile (USACH), Santiago 9160000, Chile; francis.gonzalez@usach.cl (F.G.); julian.quintero@usach.cl (J.Q.); 2 Laboratory of Cardiorespiratory Control, Departamento de Fisiología, Facultad de Ciencias Biológicas, Pontificia Universidad Católica de Chile (PUC), Santiago 3542000, Chile; rdelrio@bio.puc.cl; 3 Centro de Excelencia en Biomedicina de Magallanes (CEBIMA), Universidad de Magallanes, Punta Arenas 6200000, Chile

**Keywords:** sulforaphane, ethanol extraction, broccoli, optimization

## Abstract

Sulforaphane (SFN) is a powerful health-promoting compound found in broccoli in the form of its inactive precursor, glucoraphanin (GFN). SFN formation occurs through the enzymatic hydrolysis of glucoraphanin by myrosinase under specific chemical conditions. Its incorporation in food formulations has been hindered by the thermal instability of SFN and low concentration in *Brassicaceae*. Then, extracting SFN from broccoli at a temperature below 40 °C appears as an option to recover and stabilize SFN, aiming at delivering it as a nutraceutical. We studied an eco-friendly extraction process to obtain an SFN-rich extract from broccoli. The effect of the broccoli mass/solvent ratio, ethanol concentration in the extractant solution, and extraction time on the recovery of SFN, GFN, phenolic compounds, and antioxidant activity were studied through a Box–Behnken design. The regression models explained more than 70% of the variability in the responses, adequately representing the system. The experimental factors differently affected the bioactive compound recovery and antioxidant activity of the extracts. The extraction conditions that allowed the highest recovery of bioactive compounds and antioxidant activity were identified and experimentally validated. The results may provide the basis for the design of a process to produce a sulforaphane-rich food supplement or nutraceutical by using a GRAS extractant.

## 1. Introduction

*Brassicaceae* plants, such as broccoli, constitute a crop of nutritional and economic relevance worldwide. Global broccoli production has increased by around 30% since 2005 (https://www.tridge.com/). This relates, at least in part, to the growing demand for healthy foods. Broccoli exhibits several functional properties associated with secondary metabolites such as phenolic compounds, carotenoids, chlorophyll, alkaloids, and glucosinolates, among others [1]. In recent years, sulforaphane (SFN), a broccoli secondary metabolite, has gained great attention. SFN is an isothiocyanate found in the *Brassicaceae* family, such as broccoli (*Brassica oleracea* var. *italica*). It is recognized as a powerful anticancer and health-promoting compound naturally occurring in food [2]. Its precursor glucosinolate is glucoraphanin (GFN), which is found mainly in broccoli, and therefore, this vegetable is the main source of SFN. Domestic processing negatively affects some bioactive properties of broccoli, among them GFN and SFN content. Different efforts have been conducted to minimize the nutritional detriment of broccoli and even improve it during processing. Accordingly, it has been possible to significantly increase the content of SFN and other bioactive compounds, such as seleno amino acids [3] and polyphenols [4]. Indeed, we recently developed a strategy to maximize SFN content in the edible parts of broccoli. The process consisted of blanching at 57 °C for 13 min [5], incubation at 38 °C for 1 h [6], and freeze-drying [7]. Overall, it was possible to increase the initial SFN content by 10-fold. Unfortunately, SFN is a thermolabile molecule, which makes it difficult to include the SFN-rich broccoli powder into further elaborated foods. Thus, an option to exploit the health-promoting properties of SFN is to extract it from broccoli. This could help to stabilize the compound and minimize thermal processing. 

On the other hand, broccoli culture results in about 15% of heads that do not meet commercial standards due to excessive size and heterogeneous shape. This represents economic losses that could be reduced by processing the discarded heads, for instance by extracting bioactive compounds valuable in the food industry.

Information about the extraction processes of phytochemicals from broccoli is scarce. The extraction technique for recovering bioactive compounds from *Brassicaceae* has been suggested as a bottleneck [8]. At the analytical level, SFN is extracted from broccoli by grinding, followed by immersion in methylene chloride under agitation or aided by ultrasound or microwave processing [9]. This solvent is not recommended for its use at the production level because of its toxicity and high cost. Additionally, SFN has been extracted from *Brassicaceae* seeds using ethyl acetate as solvent [10], a generally recognized as safe (GRAS) solvent by the Food and Drug Administration (U.S.A.). Ethyl acetate is inexpensive, but it is highly inflammable, representing risks during processing. In the food industry, this solvent is used in sweets, confectionery, and soft drinks because of its fruity flavor. Accordingly, its use in salty foods is limited. García-Saldaña et al. [11] reported a separation and purification process to obtain SFN from broccoli seeds. The process consisted of chromatographic separation of an extract obtained by ultrasound-assisted ethyl acetate extraction. Xing et al. [12] reported that high-Pressure homogenization before extraction with ethyl acetate improved SFN recovery by 3-fold from broccoli seeds. Because SFN is a hydrophobic molecule, its solubility in ethanol is higher than in pure water, and therefore, ethanol could be an alternative to ethyl acetate and methylene chloride for SFN extraction from broccoli. Ethanol is GRAS and relatively inexpensive, and its inflammation risk is lower than ethyl acetate. Additionally, SFN stability in ethanol is significantly higher than in methanol, water, and methanol/water solution [13].

Jaiswal et al. [14] studied the effect of extractant solvent on the recovery of bioactive compounds and antioxidant properties from broccoli florets. The authors reported that a 60% ethanol solution allowed a higher recovery of phenolic compounds and antioxidant properties than a 60% acetone solution. However, the recovery obtained with 60% methanol was higher. This work did not consider SFN and/or GFN recovery. 

No reports about the recovery of SFN by ethanol extraction from broccoli florets are currently available. Accordingly, the main aim of this work was to investigate a scalable extraction process to obtain an SFN-rich extract from broccoli florets that could be used in the food industry. Therefore, the recovery of phenolic compounds and antioxidant properties were analyzed.

## 2. Results

The responses of each experimental run are given in Table 1. In most runs, there was a significant decrease in the content of SFN, GFN, and total polyphenols (TPP) content, as well as a reduction in the antioxidant activity, in comparison with nonextracted broccoli. The highest SFN content in the extract was 682.9 ± 46.1 µg/g dw, obtained with a solid/liquid ratio of 1:30, 80% ethanol, and 70 min extraction (Run 8). The highest GFN content was 40.1 ± 3.3 µg/g dw, obtained with a solid/liquid ratio of 1:10, 80% ethanol, and 40 min extraction (Run 4). Run 3 gave the highest TPP (460.5 ± 9.6 mg GAE/100 g dw) and FRSA (1451.4 ± 9.5 TE/100 g dw), with a solid/liquid ratio of 1:50, 80% ethanol, and 40 min extraction. Finally, the highest FRAP was attained in Run 7 (167.0 ± 4.5 TE/100 g dw), using a solid/liquid ratio of 1:30, 0% ethanol, and 70 min extraction. The maximum SFN recovery was 69.4% of the content in broccoli florets, the maximum GFN recovery was 56.6%, and the maximum TPP recovery was 100%. FRAP and FRSA showed significant differences with broccoli florets, except in Runs 3 and 7 (FRAP) and Run 9 (FRSA).

**Figure 1:**
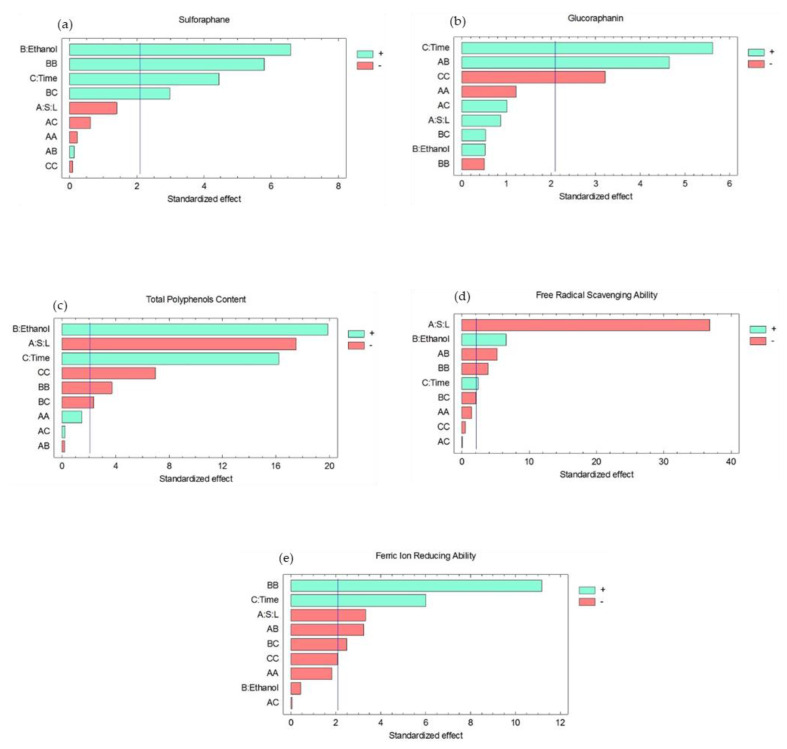
Standardized effects of the experimental factors on (**a**) sulforaphane, (**b**) glucoraphanin, (**c**) total polyphenols content, (**d**) free radical scavenging ability, and (**e**) ferric-ion-reducing ability. S:L means solid-to-liquid ratio. In the statistical analysis, the factor “A” refers to solid-to-liquid ratio, “B” refers to ethanol concentration in the extracting solution, and “C” refers to extraction time.

Figure 1 shows the significance of the statistical effects of the experimental factors on the responses. Extraction time had a significant positive effect on all responses, as well as ethanol concentration, except on FRAP, which was not significantly affected by ethanol concentration. The solid/liquid ratio had a significant negative effect on antioxidant properties and TPP in the extract, while this factor had a significant positive effect on GFN content. The solid/liquid ratio had no significant effect on SFN.

**Figure 2:**
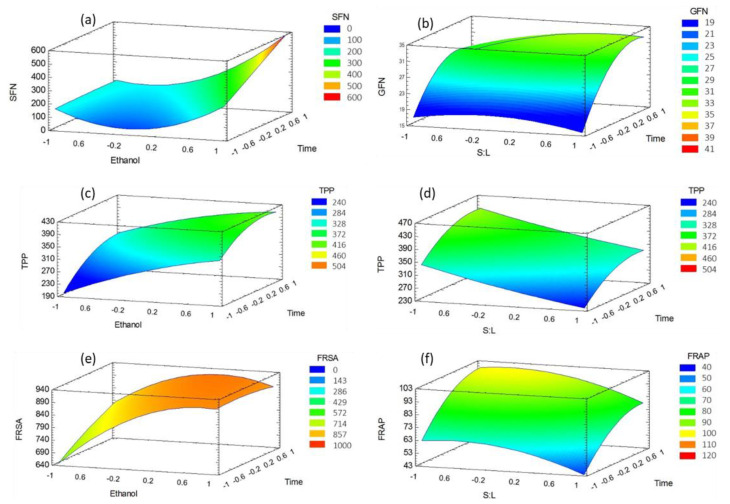
Response surfaces obtained for (**a**) sulforaphane, (**b**) glucoraphanin, (**c**,**d**) total polyphenols content, (**e**) free radical scavenging ability, and (**f**) ferric-ion-reducing ability.

Figure 2 shows the response surfaces described by the regression models presented in Table 2 for each response. Surfaces obtained for GFN, TPP (ethanol–time), FRSA, and FRAP suggest the existence of a maximum in the experimental space. The SFN response surface suggests the existence of a minimum in the experimental space. No maximum was detected for SFN, where the highest recoveries were achieved in a vertex of the surface.

Table 3 shows the validation of the regression models obtained for each response variable. Validation consisted of conducting extraction processes in the optimal conditions predicted by the regression models and experimentally determining the recovery of SFN, GFN, TPP, and antioxidant activity. Deviation of the predicted values with respect to the measured variables was calculated by means of Equation (6).

(6) $$\mathrm{Deviation}\;=\;\sqrt{{\left(\frac{predicted\;-meassured}{predicted}\right)}^{2}}\times 100\;\left[\%\right]$$

## 3. Discussion

### 3.1. Effect of the Experimental Factors on Bioactive Molecules

SFN content in pretreated broccoli before extraction was around 14-fold higher than GFN content (Table 1). In fresh broccoli, this relation is inverse, with GFN content being around 12-fold higher than SFN content [5]. This confirms that the preprocessing was effective in increasing SFN content in broccoli florets before extraction, as conversion of GFN to SFN was maximized.

The highest recoveries of bioactive compounds were achieved with 80% ethanol extracting solution due to the higher solubility of SFN, GFN, and phenolic compounds in ethanol compared to pure water. This agrees with the authors of [15], who reported SFN solubility in water is 2.55 mg/mL, while in ethanol it is 20 mg/mL. Nga [16] reported that the recovery of glucosinolates from white cabbage in 70% ethanol was around 5.8-fold higher than in water, but it was only 75% of the recovery in methanol. Glucosinolates are usually recovered from plant tissues together with phenolic compounds [17], then both kinds of molecules have similar behavior in alcohol/water solutions. Do et al. [18] studied the recovery of phenolic compounds from lyophilized *L. aromatic* in different solvents, reporting recoveries in 75% ethanol and in 75% methanol of around 5-fold higher than recovery in pure water. The similar recoveries in different solvents suggest that phenolic compounds were totally recovered from the food matrix. This is confirmed by the 100% recovery of TPP in 80% ethanol obtained in the present work. Recovery of SFN, GFN, and TPP increases with increasing content of alcohol in the extracting solution. This agrees with the statistical analysis that indicates a significant positive effect of ethanol concentration on the recovery rates of SFN, GFN, and TPP (Figure 1). 

The highest SFN and GFN recoveries were 69.4% and 56.6%, away from 100%, probably due to the different solvents used in the analytical determinations. SFN solubility in dichloromethane was 25 mg/mL, while in ethanol it was 20 mg/mL [15]. Accordingly, SFN recovery in ethanol solution was lower than in dichloromethane. The case of GFN is similar, as analytical extraction used 70% methanol, and GFN recovery in methanol solutions was higher than in ethanol [16]. 

Extraction time had a significant positive effect on SFN, GFN, and TPP content; however, only the highest recovery of SFN was attained at the highest extraction time, unlike GFN and TPP, which reached the highest recoveries after 40 min extraction. This may be related to the statistical design, as even though it is an optimization design, it does not include all the possible level combinations. This is confirmed by the predictions of the regression model, which suggest that the optimal extraction time to maximize bioactive compounds recovery is near 70 min, as shown in Table 3.

The solid/liquid ratio had a significant negative effect on TPP content, FRSA, and FRAP. This concordant behavior is related to the fact that TPP are responsible for the antioxidant activity detected in vitro, and therefore, experimental factors affect these variables in the same way. The solid/liquid ratio did not significantly affect SFN and GFN recovery in the extract. These different behaviors may be related to the mass transfer hindrances that affect the compounds, associated with the cellular location of SFN, GFN, and TPP. TPP are ubiquitously distributed in plant cells. They are found in cell walls, vacuoles, and nuclei [19]. As a consequence, their transfer from plant tissue to the surrounding solution during extraction has different barriers. Additionally, a larger concentration gradient improves mass transfer. On the other hand, phenolic compounds are highly soluble in aqueous and alcoholic solutions; thus, saturation of the extractant solution could occur when using higher solid/liquid ratios. GFN is located in the cytoplasm of glucosinolate-containing cells [20], and then after preprocessing broccoli pieces, which disrupts broccoli cells, glucosinolates become more easily extractable. Additionally, because glucosinolate solubility is lower than that of phenolic compounds, the extracting solution would not saturate in GFN. Finally, SFN and GFN concentrations are comparatively lower than that of TPP; thus, the concentration gradient is larger for SFN and GFN during extraction. The solid/liquid ratio had no significant effect on SFN and GFN recovery probably because the effects of the other factors were considerably higher in comparison with that of the solid/liquid ratio. 

### 3.2. Effect of the Experimental Factors on Antioxidant Activity

Extraction time significantly affected the antioxidant capacity of the extracts in a positive way (FRSA and FRAP), while the solid/liquid ratio significantly affected both in a negative way. This is related to the larger concentration gradient when extracting a lower amount of broccoli tissue. Ethanol concentration had a significant positive effect on FRSA, but it did not affect FRAP. These differences can be related to the variety of compounds responsible for antioxidant activity, such as ascorbic acid, carotenoids, and flavonoids, among others, which vary in solubility and have different mechanisms to neutralize oxidant compounds. This agrees with the authors of [21], who reported the correlation between individual antioxidant molecules found in *Brassicaceae* and antioxidant activity. The authors found determination coefficients lower than 0.7, with the highest correlation obtained for polyphenols. This is confirmed by our results, as the highest TPP content was obtained using the same extraction conditions as FRAP (Run 3 in Table 1), but this does not agree with the maximum FRSA (Run 7). These results also confirm that the detected action of antioxidant compounds depends on the analytical method used to quantify antioxidant activity.

### 3.3. Optimization of the Extraction Conditions

The data were analyzed by surface response methodology. Each surface (Figure 2) was represented with the regression models shown in Table 2, and the optimum conditions were obtained from the corresponding model. The models consider only the statistically significant factors. 

The determination coefficients (R^2^) of the models were higher than 70%, indicating that the experimental factors explain 70% or more of the variability in the responses. The highest R^2^ (above 90%) was obtained for responses significantly affected by a higher number of factors because the models have fewer degrees of freedom. The models obtained for SFN (R^2^ = 82.5%) and FRAP (R^2^ = 87.8%) could probably be improved by considering additional factors in the experimental design. Temperature most likely plays a relevant role in extraction because it determines solubility, but it was not included in this study because SFN is a highly thermolabile molecule, and a high temperature (above 40°) would have altered the statistical analysis. 

The models predicted different optimal conditions for the different responses; therefore, each response was optimized individually (Table 3). Deviations between predicted and experimentally measured values were below 10% for SFN and FRSA, while the highest deviation was obtained for GFN, whose model showed the lowest determination coefficient. Accordingly, the models give acceptable predictions to define the extraction conditions that maximize the recovery of bioactive compounds and antioxidant activity. 

## 4. Materials and Methods

### 4.1. Plant Material

Broccoli (*Brassica oleracea* var. *italica* cv. Imperial) heads (three days after harvesting) were purchased from the local market (Santiago, Chile) from a single supplier. Broccoli heads were washed and cut into single florets of 5 cm length and 0.7–0.9 cm width (stem). Then, broccoli pieces were blanched using the optimal conditions determined by [5,6], which allow the conversion of GFN into SFN of up to 94%. Briefly, broccoli florets (300 g) were immersed in 1.5 L of deionized water in a thermostatic bath (Stuart, United Kingdom, Great Britain) at 57 °C for 13 min. In these conditions, epithiospecifier protein, which favors the conversion of SFN into undesirable epithionitriles, is inactivated, while myrosinase, stable at up to 70 °C and higher, remains active. After blanching, broccoli florets were withdrawn from the thermostatic bath and cooled at ambient temperature. Broccoli pieces were crushed in a screw mill to obtain 0.5 cm broccoli pieces, placed in a capped glass bottle, and incubated in a water bath at 38 °C for 60 min. This step allows GFN hydrolysis at the optimal temperature of myrosinase during a period of time in which the reaction proceeds. 

### 4.2. Experimental Design

The system was studied through the surface response methodology using a Box–Behnken design with two replicates. The experimental factors (and levels) were broccoli mass/solvent volume ratio (1:50; 1:30, and 1:10), ethanol concentration in the extractant solution (0, 40, and 80%), and extraction time (10, 40, and 70 min). The responses were the content of SFN, glucoraphanin (GFN), total polyphenols (TPP), free radical scavenging ability (FRSA), and ferric-ion-reducing ability in the extract. Extracts were obtained by immersing the broccoli mass stated by the experimental design in 50 mL of the solvent mixture under permanent agitation. After the defined time, the mixture was centrifuged, and the supernatant was recovered for further analysis.

### 4.3. Statistical Analysis

The statistical effects of the experimental factors on the responses were determined using a second-order polynomial model to describe the experimental behavior (Equation (7)).

(7) $$Y=\beta_{0}+\overset{k}{\underset{i=1}{\sum }}\beta_{i}x_{i}+\overset{k}{\underset{i=1}{\sum }}\beta_{ii}x_{i}^{2}+\sum^{\;}\overset{k}{\underset{i<j=1}{\sum }}\beta_{ij}x_{i}x_{j}$$

where *Y* is the predicted value of the response; $\beta_{0}$, $\beta_{i}$, $\beta_{ii}$, and $\beta_{ij}$ are the regression coefficients for intercept, lineal, quadratic and interaction effects, respectively; k is the number of independent parameters (k=3 in this study); and $x_{i},\;x_{j}$ are the coded levels of the experimental conditions. To determine the significant effect of factors, analysis of variance (ANOVA) at 95% confidence was applied using STATGRAPHICS^®^ Centurion XVI. The quality of the model was assessed by the determination coefficient (R^2^) and the determination coefficients adjusted by degrees of freedom (R^2^ adj).

### 4.4. Analytical Determinations

#### 4.4.1. Sulforaphane

Sulforaphane content was determined by reverse-phase HPLC using the method proposed by [9]. Broccoli samples were pulverized in a mortar, until obtaining a homogeneous meal. One gram of the sample was extracted two times with 10 mL of methylene chloride (J.T. Baker, Center Valley, PA, USA), which was combined with 0.5 g of anhydrous sodium sulfate (Sigma-Aldrich, Schnelldorf, Germany). After agitation for 60 min in a shaker, the sample was filtered, and the solution was rotavaporized at 30 °C under vacuum until dryness. For quantification in ethanolic extracts, 1 mL of extract was dried, suspended in methylene chloride, and processed as described above. The equipment was a HPLC-DAD (Shimadzu, Kyoto, Japan), and a C18 column (5 μm particle size, 250 × 4.6 mm) (Agilent Technologies, Santa Clara, CA, USA) was used. The solvent consisted of 20% acetonitrile (Merck, Darmstadt, Germany) in HPLC-grade water, changing linearly over 10 min to 60% acetonitrile and maintained at 100% acetonitrile for 5 min. The column oven temperature was set at 30 °C, the flow rate was 1 mL min^−1^, and the injection volume was 20 µL. Absorbance at 254 nm was recorded. Sulforaphane was eluted at 2.5 min. Quantification was carried out by comparison with a sulforaphane standard curve (0.056–6.75 μg). Organic solvents (HPLC grade) were purchased from Merck (Darmstadt, Germany). Sulforaphane content was expressed in μg g^−1^ (dry weight). All determinations were made in triplicate.

#### 4.4.2. Glucoraphanin Content

Glucoraphanin was quantified by reverse-phase HPLC [22]. Broccoli was pulverized, and 1.5 methanol (70%) was added to 100 mg of powder in a microcentrifuge tube. The mixture was agitated for 30 min in a water bath at 70 °C. After that, the tube was centrifuged at 13,000 rpm for 15 min, the supernatant was recovered, dried in a rotary evaporator at 30°, and the residue was resuspended in 1 mL of ultrapure water. Ethanolic extracts (1 mL) were dried at 30 °C under vacuum on a rotary evaporator, and the residue was resuspended in 1 mL of HPLC-grade water. The samples were filtered through a 0.22 µm syringe filter before injection in HPLC. The HPLC equipment was a HPLC-DAD (Shimadzu, Tokyo, Japan), and a reverse-phase C18 column (5µm particle size, 250 × 4.6 mm) (Agilent Technologies, Santa Clara, CA, USA) was used. The mobile phase was water + TFA 0.1% *v*/*v* (A) and acetonitrile + TFA 0.1% *v*/*v* (B), the gradient elution program consisted of 5 min of 100% A, then 3 min in which the solution was changed to 94.9% of A, the next 7 min reaching 99% of B, and finally 10 min of 100% A. The column temperature was set at 30 °C, and a 1 mL/min flow was used. Aliquots of 20 μL of the sample were injected into the column. The glucoraphanin peak was detected by absorption at 226 nm. Glucoraphanin was eluted at 5.0 min. Quantification was carried out by comparison with a glucoraphanin standard curve (0.01–7.00 μg). The results were expressed in μg/g dw. The determinations were made in duplicate. 

#### 4.4.3. Total Polyphenols Content

The total polyphenols were quantified spectrophotometrically through the Folin–Ciocalteu method [23]. Pulverized broccoli (200 mg) was mixed with 4 mL of methanol (80%), sonicated for 10 min at 8 V, agitated for 1 h, centrifuged at 13,000 rpm for 5 min, and flushed to 5 mL. Broccoli or ethanolic extract (180 µL) was mixed with 360 µL of HPLC-grade water and 90 µL of Folin–Ciocalteu reagent. The mixture was homogenized and left in the dark for 5 min. After that, 450 µL of 200 g/L sodium carbonate solution was added to stop the reaction and left in the dark again for 30 min. After that, samples were centrifuged at 13,000 rpm for 5 min, and the supernatant was recovered. Absorbance at 750 nm was recorded. The results were expressed as mg of gallic acid equivalent (GAE) per gram of dry weight. The measurements were made in duplicate.

#### 4.4.4. Ferric-Ion-Reducing Ability

The ferric-ion-reducing ability (FRAP) was measured following the protocol reported by [24]. FRAP reagent was obtained by mixing a solution of 20 mM FeCl3·6H2O with a solution of 10 mM TPTZ in 40 mM HCl and 0.3 M acetate buffer (pH = 3.6) in the volume proportion of 1:1:10. An aliquot extract of 100 µL was mixed with 400 µL of 80% methanol and 1 mL of FRAP reagent, agitated, and incubated at 37 °C for 30 min in the dark. Absorbance at 593 nm was recorded. All measurements were made in duplicate.

#### 4.4.5. Free Radical Scavenging Ability

The free radical scavenging ability (FRSA) was measured using the stable radical 2,2-diphenyl-1-picrylhydrazyl (DPPH) [25]. Extracts were diluted (at 6 dilutions), and 40 μL of each dilution was mixed with 1960 μL of DPPH solution (6 × 10^−5^ M in methanol). The absorbance decrease at 515 nm was continuously recorded for 30 min. The DPPH· concentration in the reaction mixture at zero time and after 30 min was calculated by means of a calibration curve, and the remaining DPPH· concentration was obtained. Results are expressed in Trolox equivalents. The measurements were made in duplicate.

#### 4.4.6. Moisture Content

The moisture content of blanched broccoli was determined according to [26]. Samples (3 g) were dehydrated at 70 °C in a vacuum oven model 60,061 (Cole-Palmer, Holdpack, IL, USA) until a constant weight was obtained. All measurements were made in duplicate.

## 5. Conclusions

The ethanol–water system can be used for recovering SFN from broccoli florets. In addition to SFN, the extracts contained significant amounts of glucoraphanin and phenolic compounds and showed high antioxidant activity, making them attractive as a food supplement but not as a source of pure SFN for pharmaceutical use. The identification of a single optimal extraction condition that simultaneously maximizes the recovery of SFN, GFN, and TPP was not possible. The conditions that allow the highest recoveries of SFN were identified as a solid/liquid ratio of 1:50, 80% ethanol in the extractant, and 70 min of extraction, recovering 56.6 mg of SFN per 100 g of broccoli (dry weight). The results obtained in this work provide the basis for the design of a process to produce a sulforaphane-rich food supplement or nutraceutical by using a GRAS extractant. The next steps of the process should include the recovery and recycling of ethanol and stabilization of the extract.

## Figures and Tables

**Table 1:** Experimental matrix. SFN = sulforaphane; GFN = glucoraphanin; TPP = total polyphenols content; FRAP = ferric-ion-reducing ability; FRSA = free radical scavenging ability; GAE = gallic acid equivalents; TE = Trolox equivalents; dw = dry weight. Maximum values are highlighted in bold. “*” indicates no statistically significant difference in broccoli (Student’s *t* test at 95% confidence).

Runs	Solid/Liquid Ratio	Ethanol (%)	Extraction Time (min)	SFN (µg/g dw)	GFN (µg/g dw)	TPP (GAE/100 g dw)	FRAP (TE/100 g dw)	FRSA (TE/100 g dw)
Preprocessed broccoli	-	-	-	**983.3 ± 3.8**	70.9 ± 6.3	458.0 ± 33.9	174.5 ± 12.1	1383.6 ± 23.5
1	−1(1:50)	−1(0)	0(40)	249.1 ± 4.5	31.3 ± 5.2	342.0 ± 7.4	132.7 ± 0.4	1018.5 ± 53.2
2	+1(1:10)	−1(0)	0(40)	136.8 ± 8.6	20.4 ± 4.3	240.6 ± 6.8	120.3 ± 1.1	316.1 ± 5.6
3	−1(1:50)	+1(80)	0(40)	429.5 ± 31.9	19.4 ± 8.3	460.5 ± 9.6 *	164.5 ± 0.1 *	**1451.4 ± 9.5**
4	+1(1:10)	+1(80)	0(40)	332.2 ± 14.9	**40.1 ± 3.3**	355.9 ± 5.2	97.5 ± 0.3	351.2 ± 2.4
5	0(1:30)	−1(0)	−1(10)	58.8 ± 4.9	19.4 ± 1.2	198.0 ±8.4	98.1 ± 1.1	657.8 ± 10.4
6	0(1:30)	+1(80)	−1(10)	213.1 ± 44.3	15.2 ± 5.2	330.5 ± 3.6	109.2 ± 0.2	853.8 ± 26.7
7	0(1:30)	−1(0)	+1(70)	207.3 ± 21.6	27.4 ± 1.1	316.4 ± 0.4	167.0 ± 4.5 *	832.3 ± 35.8
8	0(1:30)	+1(80)	+1(70)	**682.9 ± 46.1**	30.1 ± 6.1	410.2 ± 0.1	136.3 ± 2.2	872.2 ± 25.6
9	−1(1:50)	0(40)	−1(10)	91.4 ± 5.8	18.1 ± 0.1	342.5 ± 2.1	58.6 ± 3.9	1373.5 ± 3.6 *
10	+1(1:10)	0(40)	−1(10)	121.8 ± 8.9	14.9 ± 3.3	240.8 ± 0.7	59.4 ± 0.9	294.5 ± 2.3
11	−1(1:50)	0(40)	+1(70)	154.1 ± 2.0	31.9 ± 0.4	429.1 ± 7.5*	82.7 ± 3.1	1409.0 ± 17.1
12	+1(1:10)	0(40)	+1(70)	118.9 ± 11.2	32.3 ± 1.6	331.2 ± 0.4	82.5 ± 0.1	327.6 ± 1.7
13	0(1:30)	0(40)	0(40)	172.8 ± 5.1	26.3 ± 3.2	343.3 ± 5.1	80.9 ± 0.7	860.1 ± 8.2
14	0(1:30)	0(40)	0(40)	103.6 ± 12.9	37.3 ± 3.3	384.9 ± 11.2	98.8 ± 3.2	905.7 ± 2.5
15	0(1:30)	0(40)	0(40)	114.4 ± 2.1	28.9 ± 1.9	349.7 ± 4.5	83.8 ± 0.4	903.4 ±1.1

**Table 2:** Regression models for each response in coded variables. A = solid/liquid ratio; B = ethanol concentration in the extractant solution; C = extraction time. R^2^ is the determination coefficient, and R^2^ adj is the determination coefficient adjusted by degrees of freedom. SFN is sulforaphane in µg/g dw, GRA is glucoraphanin in µg/g dw, TPP is total polyphenols in mg GAE/100 g dw, FRAP is antioxidant activity measured as ferric-ion-reducing ability, and FRSA is antioxidant activity measured as free radical scavenging ability. Models consider only the statistically significant variables.

Regression Model	R^2^ (%)	R^2^ adj (%)	Equation
SFN°= 125.3 + 125.7° × *B* + 84.7° × *C* + 163.4° × *B*^2^ + 80.3° × *B*° × *C*	82.5	79.8	(1)
GFN = 29.1 + 7.9° × *A* + 6.8° × *C* + 15.8° × *A*° × *B*° − 5.5° × *C*^2^	73.3	70.2	(2)
TTP = 363.2 + 50.7 × *A* + 57.5 × *B* + 46.9 × *C* − 16.3 × *B*^2^° − 9.7 × *B* × *C*° − 30.2 × °*C*^2^	97.9	97.3	(3)
FRAP = 78.1 + 0.9 × *A* + 17.9 × *C* − 13.6 × *A* × *B* + 50.1*B*^2^ − 10.4 × *B* × *C*	87.8	85.3	(4)
FRSA = 867.7 − 495.4 × *A* + 88.0 × *B* + 32.7 × *C* − 99.4 × *A* × *C* − 73.5 × *B*^2^ − 39.1 × *B* × *C*	98.5	98.1	(5)

**Table 3:** Validation of the regression models. Deviation was calculated by Equation (6). SFN is sulforaphane, GRA is glucoraphanin, TPP is total polyphenols, FRAP is antioxidant activity measured as ferric-ion-reducing ability, and FRSA is antioxidant activity measured as free radical scavenging ability. Factor levels are given in real variables, and the coded levels are given in parentheses.

Response	Optimal Conditions			Predicted by Regression Model	Measured Experimentally at the Optimal Conditions	Deviation (%)
	Solid/Liquid Ratio	Ethanol (%)	Time (min)			
SFN (µg/g dw)	1:50 (−1)	80 (+1)	70 (+1)	579.5	565.9 ± 8.6	0.9–3.8
GFN (µg/g dw)	1:50 (−1)	40 (0)	58 (+0.60)	39.1	46.8 ± 3.1	11.8–27.6
TPP (GAE/100 g dw)	1:50 (−1)	80 (+1)	58 (+0.6)	466.3	405.4 ± 19.9	8.8–17.3
FRAP (TE/100 g dw)	1:10 (+1)	0 (−1)	70 (+1)	156.9	127.2 ± 0.1	18.8–19.0
FRSA (TE/100 g dw)	1:50 (−1)	80 (+1)	10 (−1)	1483.3	1405.3 ± 28.0	3.4–7.2

## Data Availability

Not applicable.

## Sample Availability

Samples of the compounds are not available from the authors.
